# Identifying Antibacterial Compounds in Black Walnuts (*Juglans nigra*) Using a Metabolomics Approach

**DOI:** 10.3390/metabo8040058

**Published:** 2018-09-29

**Authors:** Khanh-Van Ho, Zhentian Lei, Lloyd W. Sumner, Mark V. Coggeshall, Hsin-Yeh Hsieh, George C. Stewart, Chung-Ho Lin

**Affiliations:** 1 The Center for Agroforestry, School of Natural Resources, University of Missouri, Columbia, MO 65211, USA; vkh6c6@mail.missouri.edu; 2 Department of Food Technology, Can Tho University, Can Tho 90000, Vietnam; 3 Metabolomics Center, University of Missouri, Columbia, MO 65211, USA; leiz@missouri.edu (Z.L.); sumnerlw@missouri.edu (L.W.S.); 4 Department of Biochemistry, Bond Life Sciences Center, University of Missouri, Columbia, MO 65211, USA; 5 U. S. Northern Research Station, USDA-Forest Service, West Lafayette, IN 47907, USA; coggeshallm@missouri.edu; 6 Department of Veterinary Pathobiology, Bond Life Sciences Center, University of Missouri, Columbia, MO 65211, USA; hsiehh@missouri.edu (H.-Y.H.), stewartgc@missouri.edu (G.C.S.)

**Keywords:** black walnut, *Juglans nigra*, antibacterial, metabolomics approach, compound identification

## Abstract

Black walnut (*Juglans nigra* L.) is one of the most economically valuable hardwood species and a high value tree for edible nut production in the United States. Although consumption of black walnut has been linked to multiple health-promoting effects (e.g., antioxidant, antimicrobial, anti-inflammatory), the bioactive compounds have not been systematically characterized. In addition, the associations between different black walnut cultivars and their health-promoting compounds have not been well established. In this study, the kernels of twenty-two black walnut cultivars selected for nut production by the University of Missouri Center for Agroforestry (Columbia, MO, USA) were evaluated for their antibacterial activities using agar-well diffusion assay. Among the selected cultivars, four black walnut cultivars (i.e., Mystry, Surprise, D.34, and A.36) exhibited antibacterial activity against a Gram-positive bacterium (*Staphylococcus aureus*), whereas other cultivars showed no effect on the inhibition of this bacterium. The antibacterial compounds showing the strongest activity were isolated with bioassay-guided purification and identified using a metabolomics approach. Six antibacterial bioactive compounds responsible for antimicrobial activity were successfully identified. Glansreginin A, azelaic acid, quercetin, and eriodictyol-7-*O*-glucoside are novel antibacterial compounds identified in the kernels of black walnuts. The metabolomics approach provides a simple and cost-effective tool for bioactive compound identification.

## 1. Introduction

Black walnut (*Juglans nigra* L.), known as eastern black walnut or America walnut, is economically valuable tree for hardwood and nut production [1,2], distributed throughout most of the eastern half of the United States [3]. The value of black walnut growing stock on timberland is estimated to be over one-half trillion dollars in the eastern United States [4]. This species is the second highest produced walnut nut in the United States and Missouri is the leading producer of black walnut [5]. Black walnut is often preferred in the food industry due to its unique flavor and aroma.

Black walnut has been identified and selected for propagation since late 1800s [6]. Currently, over 700 black walnut cultivars have been recorded and selected for either timber and nut production during the past century [7,8]. Selection traits for black walnut orchards include several characteristics such as yield, percent kernel, cultivar traits leafing date, flowering dates, growth habit, disease resistance, precocity, productivity, and shelling quality [9]. Several black walnut cultivars have been selected for nut production by the University of Missouri (MU) Center for Agroforestry (Columbia, MO, USA) [10].

Black walnut is an excellent source for phytochemical compounds including phenolic acids, flavonoids, and catechins [11] and monounsaturated fatty acids [5]. Our previous studies have revealed that the kernel of black walnuts contain several bioactive compounds such as quinic acid, gallic acid, *p*-hydroxybenzoic acid, vanillic acid, syringic acid, quercetin-3-d-glucoside, epicatechin gallate, rutin, naringin, and ferulic acid [11]. These compounds have been successfully identified from eleven black walnut cultivars using liquid chromatography-tandem mass spectrometry (LC-MS/MS) analysis [11]. In fact, the kernel extraction of black walnuts has been linked to antibacterial properties [12,13,14,15,16,17,18]. The stem bark extraction of English walnut (*Juglans regia* L.) has been reported to exhibit the antibacterial activity against methicillin-resistant *Staphylococcus aureus* [19]. However, the antibacterial activities among the extracts from different black walnut cultivars have never been compared, and predominant bioactive compounds in black walnut have not been isolated or characterized.

Reverse-phase flash chromatography has been widely utilized to fractionate and separate the biomolecules [20]. In this technique, the powder resins collated with the sample extraction are loaded on chromatography column packed resin coated with hydrophobic functional groups, such as C18 or C18 Bondesil, and connected to a fraction collector. The phytochemical compounds from the plant extraction are separated based on differences in their physicochemical properties (e.g., hydrophobicity and affinity). The phytochemical compounds migrate through the column at different rates and then are automatically collected at different times. The identification of bioactive compounds can be facilitated via phytochemical screening assays. This technique provides several advantages (e.g., reproducibility) compared to the traditional normal-phase chromatography technique.

For the current paper, we first evaluated and compared the antibacterial properties among the extracts from twenty-two black walnut cultivars selected for nut production. We then isolated and identified bioactive compounds in the kernels of the best cultivar using bioassay-guided purification followed by a metabolomics analysis.

## 2. Results 

### 2.1. Antibacterial Activity from Twenty-Two Black Walnut Cultivars

**Figure 1:**
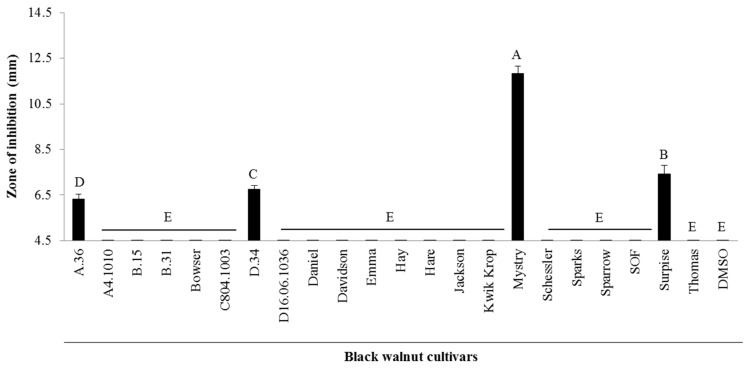
Zones of inhibition of crude kernel extracts of twenty-two black walnut cultivars grown in Missouri for *Staphylococcus aureus*. If no inhibition was observed in samples, a value of 4.5 mm, which was the diameter of the wells in agar-well diffusion experiments, was assigned. Means within bars followed by different letters are significantly different (α = 0.05, ANOVA). Mean ± SEM.

The zones of inhibition caused by kernel extracts from twenty-two black walnut cultivars against a Gram-positive bacterium (*S. aureus*) showed significant differences (*p* < 0.0001, *F*_22,75_ = 200.19) (Figure 1). Four black walnut cultivars (i.e., Mystry, Surprise, D.34, and A.36) exhibited antibacterial activity against the Gram-positive bacterium whereas other cultivars (i.e., A4.1010, B.15, B.31, Bowser, C8.04.1003, D16.06.1036, Daniel, Davidson, Emma, Hay, Hare, Jackson, Kwik Krop, Schessler, Sparks, Sparrow, South Fork, and Thomas) had no inhibitory effects on this bacterium. Mystry had the greatest zone of inhibition (11.83 ± 0.75 mm) compared to other cultivars and the zones of inhibition of Surprise, D.34, and A.36 which were 7.42 ± 0.92 mm, 6.75 ± 0.42 mm, and 6.33 ± 0.52 mm, respectively, were significantly different.

### 2.2. Identification of Antibacterial Compounds Derived from the Kernel Extract of Mystry

#### 2.2.1. Column Chromatography

**Figure 2:**
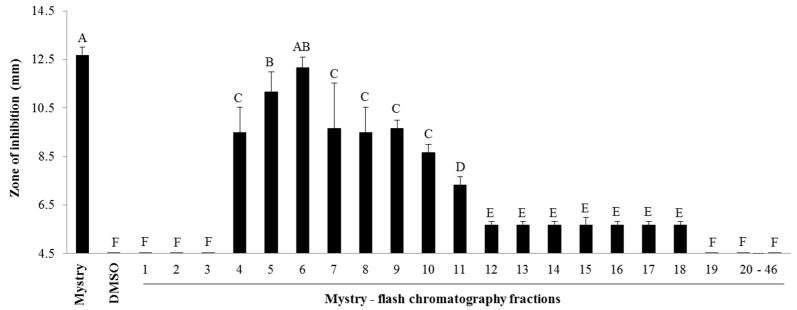
Zones of inhibition of 46 Mystry fractions from column chromatography. 20–46: fraction 20 through fraction 46. If no inhibition was observed in samples, a value of 4.5 mm, which was the diameter of the wells in agar-well diffusion experiments, was assigned. Means within bars followed by different letters are significantly different (α = 0.05, ANOVA). Mean ± SEM.

The fractionation of kernel extract from Mystry by column chromatography yielded 46 fractions. The antibacterial activities of these fractions against the Gram-positive bacterium (*S. aureus*) were significant different (*p* < 0.001, *F*_47,94_ = 37.28) (Figure 2 and Supplementary Figure S1). The fraction numbers from 4 to 18 had antibacterial activities against *S. aureus* while other fractions had no inhibitory effects on *S. aureus*. The zones of inhibition of fraction 5 and 6 (11.17 ± 0.83 mm and 12.17 ± 0.44 mm, respectively) were significantly higher compared to that of other fractions. No significant difference was seen between the zones of inhibition of fraction 6 and the crude extract of Mystry whereas the zone of inhibition of fraction 5 was lower than that of the extract of Mystry (Figure 2).

#### 2.2.2. HPLC Analysis

**Figure 3:**
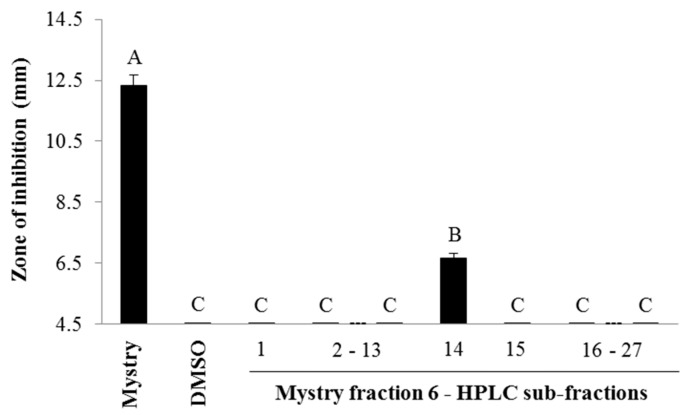
Zones of inhibition of 27 Mystry HPLC sub-fractions of fraction 6 from column chromatography. 2–13, 16–27: fraction 2 through fraction 13 and fraction 16 through fraction 27, respectively. If no inhibition was observed in samples, a value of 4.5 mm, which was the diameter of the wells in agar-well diffusion experiments, was assigned. Means within bars followed by different letters are significantly different (α = 0.05, ANOVA). Mean ± SEM.

The further separation of fraction 6 by HPLC resulted in 27 sub-fractions (Figure S2). Only sub-fraction 14 exhibited the antibacterial activity against *S. aureus* while other sub-fractions had no effect on the inhibition of the Gram-positive bacterium (Figure 3). The zone of inhibition of sub-fraction 14 was significantly higher compared to other sub-fractions, but was lower compared to the crude extract of kernel from Mystry (*p* < 0.0001, *F*_28,56_ = 466.87).

#### 2.2.3. UHPLC-QTOF-MS/MS Analysis to Identifying the Bioactive Compounds

**Figure 4:**
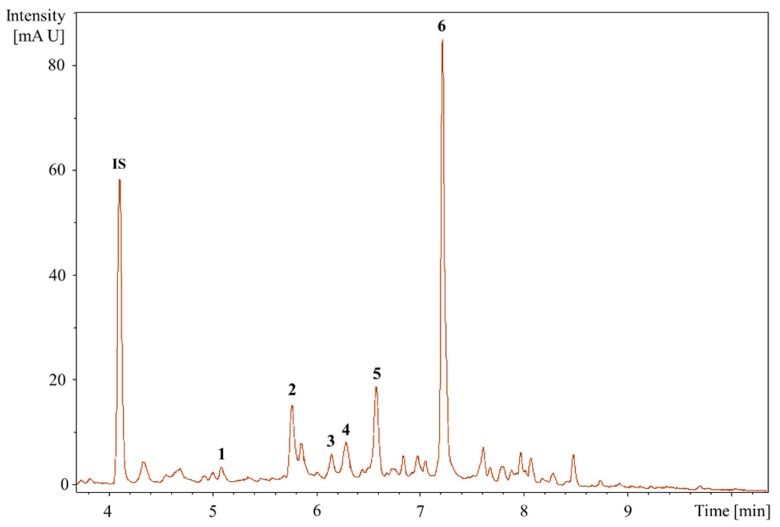
UV chromatogram of Mystry HPLC sub-fraction at 280 nm. 1–6: annotated metabolites, 1: quercetin-3-*O*-glucoside, 2: a catechin derivative, 3: eriodictyol-7-*O*-glucoside, 4: quercitrin, 5: azelaic acid, 6: glansreginin A, and IS: internal standard.

**Figure 5:**
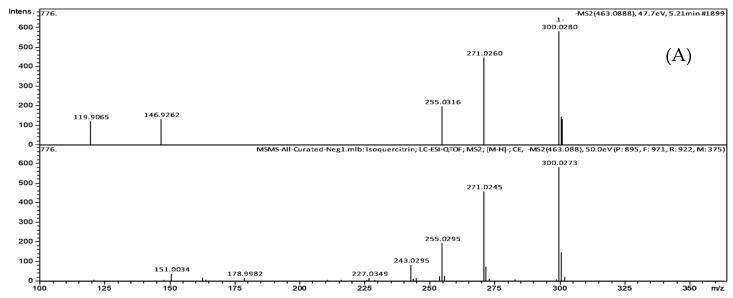

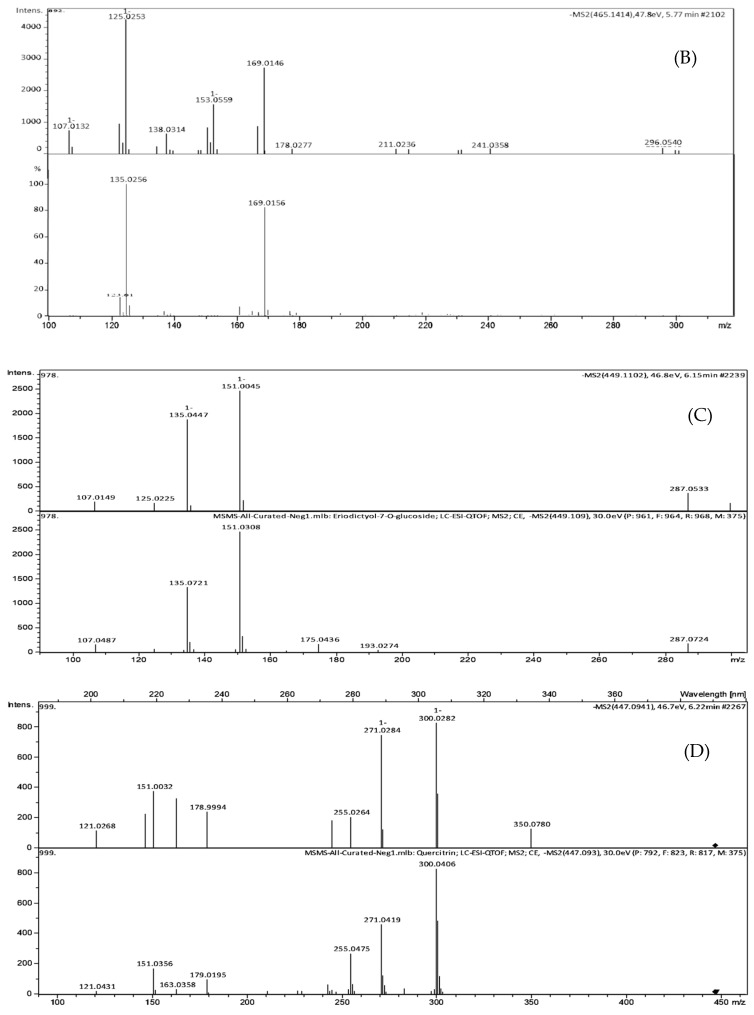

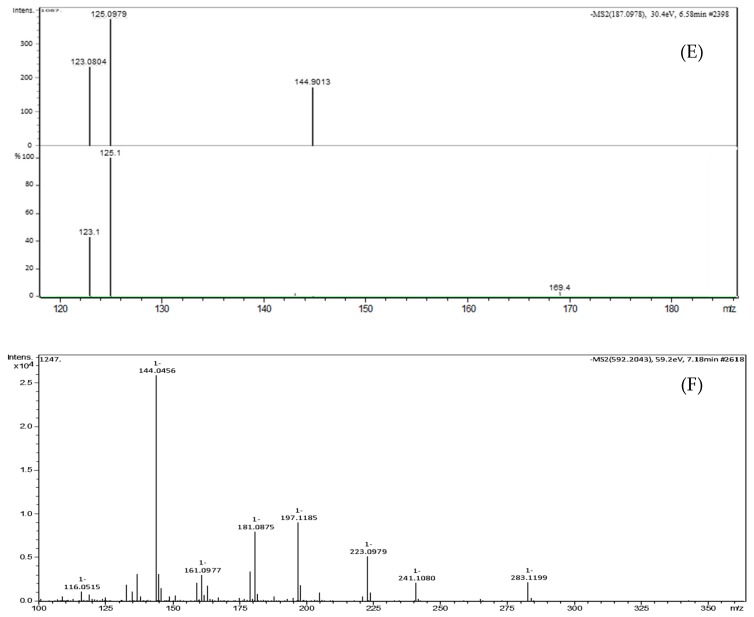
Head-to-tail spectral comparisons between the experimental MS/MS spectra and the referenced library MS/MS spectra. (**A**) Peak 1: Isoquercitrin, (**B**) peak 2: a catechin derivative; (**C**) peak 3: eriodictyol-7-*O*-glucoside, (**D**) peak 4: quercitrin, (**E**) peak 5: azelaic acid, (**F**) peak 6: glansreginin A. In each comparison, the experimental MS/MS spectra (upper) and the referenced library MS/MS spectra (lower). Since compound 6 was tentatively identified by MetFrag in silico interpretation of the MS/MS spectrum, head-to-tail of this spectral comparison did not show.

Fraction 14 from HPLC fractionation that showed inhibition activity was subject to UHPLC-QTOF-MS/MS analysis in both negative and positive electrospray ionization modes. Six major peaks resolved bioactive compounds were identified tentatively by searching their MS/MS spectra with the metabolomics databases (Figure 4 and Figure 5, Table 1). 

Compound **1** has retention time (rt) and *m*/*z* at 5.21 min and 463.0388, respectively. The MS/MS spectrum displayed fragment ions at *m*/*z* 301 [M − H − 162]^−^, 300 [M − H − 162 − H]^−^, 271 [M − H − 162 − 30]^−^, 255 [M − H − 162 − 46]^−^, 146 [M − H − 162 − 155]^−^, and 119 [M − H − 162 − 182]^−^. Loss of 162 suggested that it is glycosylated compound and fragment at *m*/*z* of 301 is the aglycone ion. The compound is not methylated as no loss of 15 Da was observed. The MS/MS was matched to quercetin-3-*O*-glucoside with a score of 834 (out of 1000) in the MassBank library. Thus, the compound is tentatively identified as quercetin-3-*O*-glucoside.

Compound **2** has rt and *m*/*z* at 5.77 min and 465.1414, respectively. The MS/MS spectrum displayed fragment ions at *m*/*z* of 301 [M − H − 164]^−^, 300 [M − H − 164 − H]^−^, 241 [M − H − 164 − 60]^−^, 169 [M − H − 164 − 132]^−^, 125 [M − H − 164 − 176]^−^, and 107 [M − H − 164 − 194]^−^. This information matches to agnuside, catechin-4-ol 3′-methyl ether 3-*O*-alpha-l-rhamnopyranoside, catechin 5-*O*-beta-d-glucopyranoside-4′-methyl ether, symplocoside (catechin 7-*O*-beta-d-glucopyranoside-3′-methyl ether) in Metlin database with the same mass tolerance (3.8 ppm). MS/MS similarity search showed that major fragments in MS/MS spectrum matched to those of epigallocatechin gallate with the score of 685, but the molecular weight did not match. Thus, it is possibly one of the catechin derivatives.

Compound **3** has rt and *m*/*z* at 6.15 min and 449.1102, respectively. The MS/MS spectrum displayed fragment ions at *m*/*z* 300 [M − H − 149]^−^, 299 [M − H − 149 − H^.^]^−^, 255 [M − H − 149 − 45]^−^, 200 [M − H − 149 − 100]^−^, 175 [M − H − 149 − 125]^−^, 151 [M − H − 149 − 149]^−^, and 135 [M − H − 149 − 165]^−^. The MS/MS was matched to eriodictyol-7-*O*-glucoside with a score of 872 (out of 1000) in the MassBank library. The ion 449.1102 *m*/*z* gave 151/135 as fragments, corresponding to the fragments previously described by Brito et al. [21] as key aglycone fragments. Thus, the compound is tentatively identified as eriodictyol-7-*O*-glucoside.

Compound **4** has rt and *m*/*z* at 6.26 min and 477.0941, respectively. The MS/MS spectrum displayed fragment ions at *m*/*z* 301 [M − H − 176]^−^, 300 [M − H − 176 − H^.^]^−^, 271 [M − H − 176 − 30]^−^, 255 [M − H − 176 − 46]^−^, 179 [M − H − 176 − 122]^−^, and 151 [M − H − 176 − 150]^−^. In MS/MS spectrum, the predominant ions at *m*/*z* 301 [M–H–176]^−^ gave the proof of same glucuronyl unit loss and fragment at *m*/*z* of 301 is the aglycone ion. The characteristic product ions at *m*/*z* of 271, 255, 179, and 151 indicate the aglycone of quercetin. On the basis of the mass spectral data, compound 4 is tentatively identified as quercetrin with a score of 823 (out of 1000) in the metabolomics library developed by Lei et al. [22].

Compound **5** has rt and *m*/*z* at 6.58 min and 187.0977, respectively. The MS/MS spectrum displayed fragment ions at *m*/*z* 144 [M − H − 43]^−^, 125 [M − H − 43 − 19]^−^, 123 [M − H − 43 − 21]^−^, and 97 [M − H − 43 − 47]^−^. The MS/MS product ion spectrum is dominated by *m*/*z* of 125, which corresponds to the combined loss of a molecule water and CO_2_ (62 Da). Further fragmentation at *m*/*z* of 125 leads to ions at *m*/*z* of 123 and 97. The MS/MS was matched to azelaic acid in the MassBank library with a score of 813 (out of 1000). Therefore, compound 5 was tentatively identified as azelaic acid.

Compound **6** has rt and *m*/*z* at 7.18 min and 592.2043, respectively. The MS/MS spectrum displayed fragment ions at *m*/*z* of 283 [M − H − 309]^−^, 241 [M − H − 309 − 42]^−^, 223 [M − H − 309 − 60]^−^, 197 [M − H − 309 − 86]^−^, 181 [M − H − 309 − 102]^−^, 144 [M − H − 309 − 139]^−^, and 137 [M − H − 309 − 146]^−^. The ion 592.2043 *m*/*z* gave 241/197 as fragments, corresponding to the fragments previously described by Gómez-Caravaca et al. [23] to be glansreginins A. Thus, compound 6 is tentatively identified as glansreginin A with score of 1.0 (peaks: 13/21 annotated/matched) via the MetFrag library.

## 3. Discussion

We demonstrated that the antibacterial properties of black walnuts against *S. aureus* were varied among tested cultivars. The four cultivars including Mystry, Surprise, D.34, and A.36 exhibited different antibacterial capacity against the Gram-positive bacterium (*S. aureus RN6390*). Other cultivars tested showed no effects on this bacterium. Several fractions of the kernel of Mystry from the column extraction showed the antibacterial activities (Figure 2), indicating the presence of multiple bioactive compounds and the possibilities of the synergy effects of these compounds that inhibited the bacterial growth. Vu et al. [11] reported differences in phenolic profiles of 11 different black walnut cultivars (e.g., Daniel, Davidson, Emma, Hay, Jackson, Kwik Krop, Mystry, Sparks, Sparrow, Schessler, Surprise, and Tomboy). Differences in the bioactive activity of black walnut cultivars are highly likely due to the differences in phytochemical profiles of these cultivars. This finding from this study clearly illustrated the differences in antibacterial properties among the black walnut cultivars, which can be used as a selection trait for improving the quality of nuts for nut production. 

**Figure 6:**
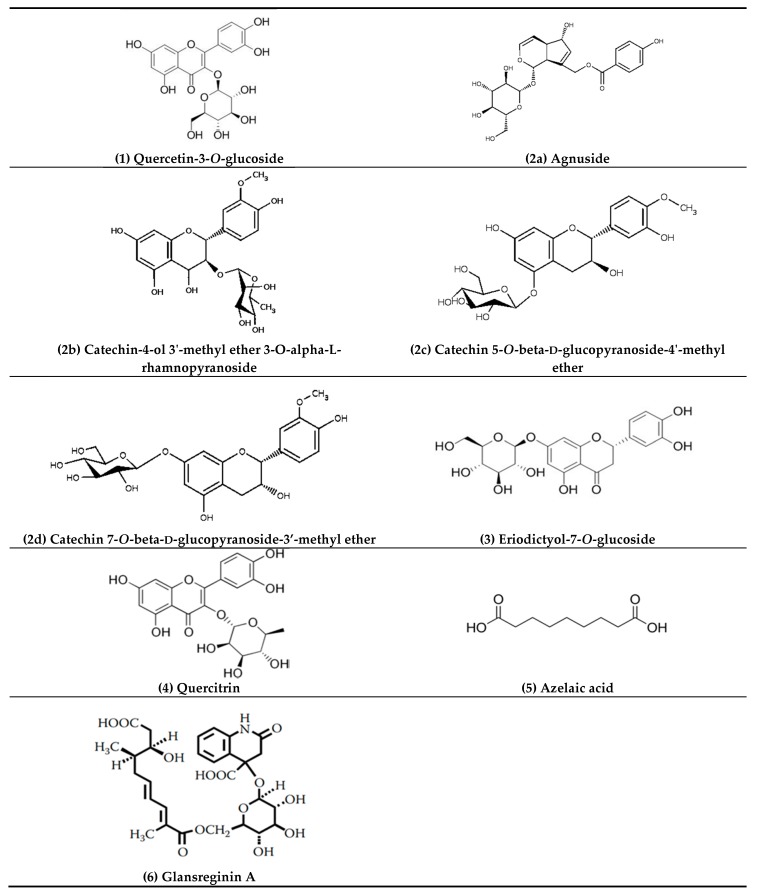
Chemical structures of tentative compounds from black walnut (Mystry).

We found six bioactive compounds (i.e., quercetin-3-*O*-glucoside, a catechin derivative, eriodictyol-7-*O*-glucoside, quercetin, azelaic acid, and glansreginin A) responsible for the antibacterial activity of the kernels of Mystry against the Gram-positive bacterium (*S. aureus*) via the metabolomics approach combined with bioassay-guided fractionation strategy (Figure 6). The information on the biological activities of these compounds is summarized in Table 2. In addition to other bioactive compounds (i.e., quinic acid, gallic acid, *p*-hydroxybenzoic acid, vanillic acid, syringic acid, isoquercetin, catechin, epicatechin gallate, rutin, naringin, ferulic acid) in the kernels of black walnuts that have been linked to possess the antibacterial properties [11], our results reveal that glansreginin A, azelaic acid, quercetin, and eriodictyol-7-*O*-glucoside are the predominant antibacterial compounds in the kernels of black walnuts according to their hydrophobicity (retention times). Among the six bioactive compounds, glansreginin A was the most abundant in the purified bioactive fraction (Figure 4). However, the analytical standard for glansreginin A is not commercially available [24], making it difficult for the validation and qualification of this compound. Future research should focus on validation and characterization of these antibacterial compounds in black walnut cultivars when the authentic standards or purified reference standards are accessible. The antimicrobial agents identified black walnut extracts could be used to prevent growth of spoilage and pathogenic microorganisms in foods. They could also be used as the natural preservatives in the formulation of personal care product.

Quercetin-3-*O*-glucoside (known as isoquercitin), a flavonoid, is widely present in a variety of plants (e.g., medicinal herbs, fruits and vegetables) such as black walnut [11], English walnut (*J. regia*) [55], and buckwheat (*Fagopyrum esculentum*) [56]. In vitro, isoquercitin has also been documented to possess antibacterial activities against several bacteria such as *S. aureus*, *S. epidermidis*, and *Propionibacterium acnes* in disk diffusion assay [25]. Many reports revealed that isoquercitin has been linked to versatile biological properties in vitro and in vivo including antioxidant [26], anti-inflammatory [27], antifungal [28], antidiabetic [29], anti-allergic [30], antitumor [31,32], antiviral [33], anti-hypertensive [34], anti-apoptotic [35], and diuretic effects [36].

Catechin derivatives (e.g., (+)-catechin, (−)-epicatechin, (−)-epigallocatechin) are found as major flavonol components in beverages, vegetables, and fruits [39,57]. Catechin, epicatechin, and epicatechin gallate have also detected previously in the kernels of black walnuts [11]. This phytochemical group has been associated with a variety of biological functions including antibacterial, antioxidant, anti-inflammatory, antiviral, and antitumor effects [37,40,41,42,57].

Eriodictyol-7-*O*-glucoside is found as a major flavonoid component derived from a Chinese herb (*Dracocephalum rupestre*), and from several plants e.g., grapevine (*Vitis vinifera*) [58], pistachios (*Pistacia vera*) [59]. The biological property of eriodictyol-7-*O*-glucoside has not been well established. Information on the biological activity of this compound has focused on the neuroprotective effect against oxidative stress in vitro and in vivo via Nrf2/ARE activation [43]. In contrast to eriodictyol-7-*O*-glucoside, eriodictyol has been associated with a variety of biological properties in vitro and in vivo such as antioxidant [60], antimicrobial [61], anti-inflammatory [62], antineoplastic [63], and antinociception [64].

Quercetin, a flavonoid, and is commonly found in vegetables (e.g., onion, garlic). The antibacterial properties against several bacteria have been also reported for quercetin [25]. Quercetin exhibited stronger antibacterial effects against three bacteria (*S. aureus*, *S. epidermidis*, and *P. acnes*) compared to isoquercetin [25]. This compound has also been linked to multiple biological functions in vitro and in vivo, such as antioxidant [25], anti-inflammatory [44], anti-allergic [30], and antitumor [65] capabilities.

Azelaic acid is a naturally occurring saturated dicarboxylic acid derived from a variety of grains such as sorghum (*Sorghum bicolor*) [66], rye (*Secale cereal*) [67]. This compound has also been reported to exert a variety of biological activities in vitro and in vivo including antimicrobial [46] and antitumor [47,48] capabilities. The U.S. Food and Drug Administration (FDA) approved 15% gel formulation of azelaic acid for the treatment of rosacea in 2002 [68] and this compound is an excellent antimicrobial agent cosmetic for the treatment of comedonal and inflammatory acnes [69,70]. In vitro, azelaic acid exhibited the antibacterial effect against *S. aureus*, *S. epidermidis*, and *P. acnes* at pH of 5.6, but no antibacterial effect was seen at pH of 7.3 [46].

Glansreginin A, a dicarboxylic acid derivative, presented dominantly in the bioactive fraction might be mainly responsible for the antibacterial activity of the kernels of Mystry. Glansreginin A has been also found in the kernels of three other nuts including English walnut [23,49,54,55], pecans (*Carya illinoinensis*) [71], and hazelnut (*Corylus avellana*) [50]. Due to the presence of this compound in the kernels of these nuts, glansreginin A has been linked to multiple biological activities in vitro and in vivo such as antioxidant [49,50,72], anti-inflammatory [51], antiatherogenic effect [52], and antinociceptive effect [53], reduction of cholesterol absorption [54]. This is the first time, glansreginin A is reported to be associated with the antibacterial properties.

In recent years, the advancement in mass spectrometry, computation power, metabolomics algorithm and mass spectral libraries in metabolomics allows rapid identification of the bioactive molecules. The metabolomics approach combined with bioassay guided fractionation strategy in this study is a promising tool for putative identification of new bioactive compounds from natural sources. The high-resolution data generated from MS/MS offer the mass accuracy and specific fragmentation fingerprints needed for rapid identification of the antimicrobial molecules. Therefore, it eliminated the time consuming and labor-intensive large-scale purification procedure that is required in the traditional structural elucidation techniques. This approach is cost-effective compared to other approaches for compound identification e.g., nuclear magnetic resonance (NMR), which typically requires at least 1 mg of the purified crystal [73].

Black walnut is an excellent resource not only for nutrition but also medicinal values. Among twenty-two black walnut cultivars tested, four cultivars exhibited antibacterial activity against the gram-positive bacterium *S. aureus* and six antibacterial compounds in the kernels of Mystry were tentatively identified. With a growing global consumption of organic personal care products and diet supplements, through identifying the novel uses of the black walnut and its byproducts, this study will provide the opportunities to turn abundant, low-value, renewable materials from the black walnut and its byproducts into profitable value-added products for the industry. Future research should focus on exploring of other health-promoting properties (e.g., antioxidant, anti-inflammatory, antitumor) and industrial applications of bioactive compounds of black walnuts through utilizing the same metabolomics strategy.

## 4. Materials and Methods

### 4.1. Black Walnut Cultivars

The nuts of twenty-two black walnut cultivars (i.e., Bowser, Daniel, Davidson, Emma, Hay, Hare, Jackson, Kwik Krop, Mystry, Schessler, Sparks, Sparrow, South Fork, Surprise, Thomas, A4, A.36, B.15, B.31, C8, D16, and D.34) were collected at the University of Missouri, Horticulture and Agroforestry Research Center, New Franklin, MO, USA. The black walnuts were hulled mechanically and hang up to dry for 15 days in a dry and darkness place at 24 °C. The hulled nuts were then stored at −20 °C until analysis.

### 4.2. Extraction of Bioactive Compounds from the Kernels of Black Walnuts

The hulled nuts were manually cracked, and the kernels were shelled and homogenized using a coffee grinder (product # CBG100S, Black + Decker, Beachwood, OH, USA) prior to extraction. The phytochemicals in the kernels of each cultivar (3 g, 20–30 mesh) were extracted with sonication in 15 mL of methanol (HPLC grade, Fisher Scientific, Pittsburg, PA, USA). The extract was sonicated for 60 min followed by centrifugation for 10 min at 4000 rpm and the supernatant was collected. Subsequently, the supernatant was filtered through a 0.2 μm Whatman Anotop syringe membrane filter (Sigma-Aldrich, St. Louis, MO, USA). The aqueous extract was evaporated until dryness under a flow of nitrogen and the final extract was resuspended with dimethyl sulfoxide (DMSO, Sigma-Aldrich, St. Louis, MO, USA) at concentration 0.12 g/mL for screening antibacterial activities using an agar-well diffusion assay.

### 4.3. Antibacterial Assay

The strain of bacteria used in this study was a Gram-positive bacterium (*S. aureus* strain RN 6390) [74]. *S. aureus* and Methicillin-resistant *S. aureus* (MRSA) are Gram-positive bacteria that are resistant to several antibiotics in the market. Identifying molecules or scaffolds that could inhibit the *S. aureus* and MRSA could help elucidating possible new antibacterial modes of action against these pathogens. In addition, many skin infections (acne) or gum disease often results from the infection of the Gram-positive bacteria. From the previous works, many antimicrobial compounds that inhibited the *S. aureus* could also use to treat skin infections and gum disease (e.g., totarol). Therefore, selecting this specific strain in the early stage screening study will help explore the future commercial application. 

The antibacterial activities of black walnut extracts were determined using agar-well diffusion assay as described by Holder and Boyce [75]. The *S. aureus* RN6390 was streaked on Luria-Bertani (LB) agar plates incubated at 37 °C for 12 h. For preparation of 1 L of LB agar, 15 g of agar (Fisher Scientific), 10 g of tryptone (Fisher Scientific), 5 g of yeast extract (Fisher Scientific) and 10 g of NaCl (Fisher Scientific) were suspended in 1 L of distilled water. The mixture was homogenized and autoclaved at 121 °C for 1 h. It was then cooled to approximately 50 °C while mixing with a magnetic stirrer. The liquid LB agar was poured into Petri plates (150 mm diameter) and allowed to solidify at 25 °C. For preparation of an culture of RN6390, the bacteria were cultured in LB broth at 37 °C for 16 h and then diluted into 5 mL LB broth (to an OD_600_ of 0.02) and incubated in a shaker at 37 °C. Once the culture reached an OD_600_ of 0.1, it was swab-inoculated onto LB agar plates. For testing antibacterial activities of the extracts, wells (4.5 mm in diameter) were cut into the surface of the agar using a cork borer. The extracts (10 µL) were pipetted into the wells and the plates then incubated under aerobic condition at 37 °C for 16 h. A biocompatible solvent (DMSO) was used as negative control. The diameters of inhibition zones were measured by a ruler, with an accuracy of 0.5 mm. Each inhibition zone was measured three times and each extract was replicated at least three times in three different plates.

### 4.4. Identification of Bioactive Compounds Using a Metabolic Approach

The black walnut cultivar that exhibited the strongest antibacterial activities against the gram-positive bacterium was selected for bioassay-guided purification. The kernel extract of this cultivar was sequentially fractionated using column chromatography (CC) and then the bioactive fraction was further fractionated using high-performance liquid chromatography (HPLC). The agar-well diffusion assay was performed to identify the antibacterial activities of the bioactive fractions. The HPLC sub-fraction that had the strongest antibacterial activities against the Gram-positive bacterium was analyzed by high-resolution mass spectrometry and the mass spectra was analyzed to identify the bioactive compounds responsible for the antibacterial activities.

#### 4.4.1. Column Chromatography 

Kernels (25 g) were collected and the phytochemicals were extracted twice with methanol (100 mL:100 mL). The extract was homogenized thoroughly using a blender (Hamilton Beach, Inc., Glen Allen, VA, USA) and was sonicated for 60 min at temperature ≤ 30 °C. The extract was filtered through filter paper (125 mm in diameter, Whatman, GE Healthcare, Chicago, IL, USA) under SPE Vacuum Manifold (Visiprep^™^ SPE Vacuum Manifold, Sigma-Aldrich, Saint Louis, MO, USA) and then was concentrated by a rotary evaporator (BUCHI Rotary Evaporator R110, Buchi, Flawil, Switzerland) under a vacuum (Buchi), yielding a greenish yellow gum (4 g). The resultant gum (4 g) was dissolved in methanol (5 mL), impregnated with 4 g of sorbents Bondesil C18 (40 µm particle size; Agilent Technologies, Santa Clara, CA, USA) and placed in the hood for 12 h until all the methanol was evaporated, which yielded powder resins coated with the extract. The C18 resins with the extract were stored at 4 °C in darkness and used within a week.

The compounds were separated and fractionated by a Biotage FlashMaster II flash chromatography connected with an ISCO Foxy 200 fraction collector. The powder resins collated with the extract (8 g) were loaded on to the top of chromatography column (3.7 cm in diameter and 13.5 cm long) packed with 34 g of C18 Bondesil resin. The running time was 300 min at a flow rate of 0.5 mL/min. The mobile phase consisted of deionized water (A) and methanol (B) and a linear gradient was optimized as follows 25% B (0–40 min), 25–50% B (40–90 min), 50–75% B (90–120 min), 75% B (120–180 min), 75–100% B (180–240 min) and 100% B (240–300 min), respectively. The elution yielded forty-six fractions collected automatically by a fraction collector. All fractions were concentrated by nitrogen evaporator and re-dissolved in DMSO at 10× concentration for antibacterial activities testing using an agar-well diffusion assay.

#### 4.4.2. HPLC Analysis

The bioactive fraction from column chromatography that exhibited the strongest antibacterial activities against the Gram-positive bacterium was further fractionated by high-performance liquid chromatography (HPLC). The bioactive fraction (50 µL) was injected into a HPLC system consisting of a Shimadzu SCL-10Avp HPLC controller (Shimadzu Co., Columbia, MD, USA), a LC-10ADvp solvent delivery system, SIL-10ADvp auto-injector, a SPD-10Avp photodiode array detector, and a FRC-1500 HPLC micro fractionation collector (Shimadzu). The elution was performed with a Columbus C8 reverse-phase column (250 mm × 4.6 mm, 5 µm particle size; Phenomenex, Torrance, CA, USA). The running time was 30 min at a flow rate of 0.5 mL/min and the signals were monitored at both 254 nm and 220 nm. The mobile phase consisted of deionized water (A) and acetonitrile (B). The elution condition was optimized as follows 10–45% B (0–16 min), 45% B (16–16.2 min), 45–80% B (16.2–17 min), 80–98% B (17–18 min), 98% B (18–19 min), 98–20% B (19–20 min), and 20% B (20–30 min), respectively. The HPLC sub-fractions were collected automatically into fraction collection vials. The fractions having the same retention time window were pooled after 50-time injections of the bioactive fraction from column chromatography. The solvent of each separated fraction was evaporated under the hood and the samples were dissolved in DMSO at 100× concentration for testing antibacterial activities using an agar-well diffusion assay. 

#### 4.4.3. UHPLC-QTOF-MS/MS Analysis

The HPLC sub-fraction that exhibited the strongest antibacterial activities was concentrated 100X in 80% methanol containing an internal standard (umbelliferone) and then the sample was analyzed by UHPLC coupled to a maXis impact quadrupole-time-of-flight mass spectrometer (Bruker Daltonics, Gmbh, Bremen, Germany). The separation was achieved on a Waters Acquity UHPLC BEH C18 column (2.1 × 100 mm, 1.7 µm particles size) using a linear gradient of 95%: 5% to 30%: 70% eluents A: B (A: 0.1% formic acid and B: acetonitrile) in 30 min. From 30–40 min, a linear gradient was as follows 70–95% B (30–33 min), 95% B (33–35 min), 95–5% B (35–36 min), and 5% B (37–40 min), respectively. The flow rate was 0.56 mL/min and the column temperature was kept at 60 °C. Mass spectrometry was performed in both negative and positive electrospray ionization modes with the nebulization gas pressure at 43.5 psi, dry gas of 12 L/min, dry temperature of 250 °C and a capillary voltage of 4000V. MS/MS mass spectral data was collected automatically using following parameters including MS full scan from 100 to 1500 *m*/*z*, 3 precursors, threshold with 10 counts, active exclusion with three spectra released after 0.15 min, collision energy depending on mass such as 35 eV at 500 Da, 50 eV at 1000 Da and 70 eV at 2000 Da. The mass spectra were auto-calibrated using sodium formate after data acquisition. 

The metabolite annotation in significant peaks in UV chromatogram of the MS/MS mass spectra was identified based on MS/MS fragmentation. The MS/MS fragments were referenced to the Metabolomics library developed by Lei et al. [20], as well as MetFrag (https://msbi.ipb-halle.de/MetFragBeta/), MassBank of North America (http://mona.fiehnlab.ucdavis.edu/spectra/), and METLIN (http://metlin.scripps.edu).

#### 4.4.4. Statistical Analysis

In antibacterial experiments, zones of inhibition of black walnut extracts were analyzed as a randomized complete block design using PROC MIXED in SAS 9.4 (SAS Institute, Cary, NC, USA). If no inhibition was observed in the samples, a value of 4.5 mm, which was the diameter of the wells used in the agar-well diffusion assay, was assigned to these treatments prior to the analysis. The black walnut extract was the fixed effect and replication was the random variable. Differences between extracts were determined using Fisher’s LSD.

## 5. Conclusions

The antibacterial properties of the kernels of black walnuts were successfully characterized. In fact, twenty-two black walnut cultivars (i.e., Bowser, Daniel, Davidson, Emma, Hay, Hare, Jackson, Kwik Krop, Mystry, Schessler, Sparks, Sparrow, South Fork, Surprise, Thomas, A4, A.36, B.15, B.31, C8, D16, and D.34) had shown differences in their antibacterial properties against the Gram-positive bacterium (*S. aureus RN6390*) and Mystry exhibited the strongest antibacterial activity. The antibacterial activity was also seen in Surprise, D.34, and A.36, but no antibacterial effect was seen in the other tested cultivars. Six possible antibacterial compounds in the kernels of Mystry were tentatively identified through the metabolomics approach combined with bioassay-guided purification. This approach is a promising tool for identifying the candidates of bioactive molecules from natural sources.

## Figures and Tables

**Table 1:** Putative bioactive compounds responsible for antibacterial activity in black walnut (Mystry).

Peak No.	T_R_ (min)	[M-H]- (*m*/*z*)	Formula	Exact Mass	Δm (ppm)	MS/MS Fragments, *m*/*z*, Intensity (%)	Putative Identification *
1	5.21	463.0888	C_21_H_20_O_12_	464.0954	2.6	301.0308 (25.3), 300.0280 (100), 271.0260 (76.8), 255.0316 (34.3), 146.9262 (23.2), 119.9065 (21.1)	Quercetin-3-*O*-glucoside
2	5.77	465.1414	C_22_H_18_O_11_	458.0849	3.8	301.0147 (2.7), 300.0280 (3.0), 241.0358 (4.2), 169.0146 (64.2), 125.0253 (100), 107.0132 (17.5)	Agnuside Catechin-4-ol 3′-methyl ether 3-*O*-alpha-l-rhamnopyranoside Catechin 5-*O*-beta-d-glucopyranoside-4′-methyl ether Catechin 7-*O*-beta-d-glucopyranoside-3′-methyl ether
3	6.15	449.1102	C_21_H_22_O_11_	450.1162	4.0	299.9964 (12.4), 298.9994 (15.3), 255.0408 (12.4), 200.8817 (11.5), 174.9541 (17.1), 151.0039 (100), 135.0444 (77.5)	Eriodictyol-7-*O*-glucoside
4	6.26	477.0941	C_21_H_20_O_11_	448.1006	1.3	301.0373 (43.2), 300.0282 (100), 271.0284 (90.3), 255.0264 (24.8), 178.9994 (29.1), 151.0032 (45.4)	Quercitrin
5	6.58	187.0977	C_9_H_14_O_4_	186.0892	3.2	144.9013 (46.2), 125.0979 (100), 123.0804 (62.4), 97.0656 (47.8)	Azelaic acid
6	7.18	592.2043	C_28_H_35_NO_13_	593.2108	2.1	283.1199 (8.5), 241.1080 (8.2), 223.0979 (19.9), 197.1185 (34.5), 181.0875 (30.8), 144.0456 (100), 137.0972 (12.1)	Glansreginin A

* Tentative identification of compounds on the basis of MS/MS mass spectra and UV spectra reported in the referred databases.

**Table 2:** Bioactivities of six bioactive compounds responsible for antibacterial activity in the kernels of black walnut (Mystry).

No.	Compound	Bioactivities	References
1	Quercetin-3-*O*-glucoside	antimicrobial	Wang et al. [25]
		antioxidant	Chang et al. [26]
		anti-inflammatory	Li et al. [27]
		anti-fungal	Yun et al. [28]
		antidiabetic	Zhang et al. [29]
		anti-allergic	Rogerio et al. [30]
		antitumor	Amado et al. [31], Chen et al. [32]
		antiviral	dos Santos et al. [33]
		anti-hypertensive	Junior et al. [34]
		anti-apoptoti	Zhu et al. [35]
		diuretic effects	Junior et al. [36]
2	Catechin derivatives	antimicrobial	Veluri et al. [37], Hara-Kudo et al. [38]
		antioxidant	Seeram et al. [39]
		anti-inflammatory	Mizushina et al. [40]
		antitumor	Cao et al. [41]
		antiviral	Song et al. [42]
3	Eriodictyol-7-*O*-glucoside	antioxidant	Jing et al. [43]
4	Quercitrin	antimicrobial	Wang et al. [25]
		antioxidant	Wang et al. [25]
		anti-inflammatory	Yang et al. [44]
		anti-allergic	Rogerio et al. [30]
		antitumor	Liu et al. [45]
5	Azelaic acid	antimicrobial	Charnock et al. [46]
		antitumor	Pan et al. [47], Breathnach [48]
6	Glansreginin A	antioxidant	Ito et al. [49], Slatnar et al. [50]
		anti-inflammatory	Papoutsi et al. [51]
		antiatherogenic effect	Berryman et al. [52]
		antinociceptive effects	Raafat [53]
		reduction of cholesterol absorption	Ren et al. [54]

## Supplementary Materials

The following are available online at http://www.mdpi.com/2218-1989/8/4/58/s1, Figure S1: Zone of inhibition of 29 Mystry fractions (out of 46) from column chromatography. 1-29: fraction 1 through fraction 29, Figure S2: Shimadzu UV Chromatogram of HPLC sub-fraction F14 at 220 nm (**A**) and 254 nm (**B**) generated from SPD-10Avp photodiode array detector, Figure S3: Head-to-tail spectral comparisons between the experimental MS/MS spectra and the referenced library MS/MS spectra. (**A**) Peak 1: Isoquercitrin, m/z of 463.0888, retention time (rt) at 5.21 min; (**B**) Peak 2; (**C**) Peak 3: Eriodictyol-7-O-glucoside, m/z of 449.1102, rt at 6.15 min; (**D**) Peak 4: Quercitrin, m/z of 477.0941, rt at 6.26 min; (**E**) Peak 5: Azelaic acid, m/z of 187.0977, rt at 6.58 min; (**F**) Peak 6: Glansreginin A, m/z of 592.2043, rt at 7.18 min.
