# Supplementary material for: Identifying Antibacterial Compounds in Black Walnuts (Juglans nigra) Using a Metabolomics Approach
Source: Metabolites. 2018 Sep 29;8(4):58. doi: 10.3390/metabo8040058 (PMC6316014; doi:10.3390/metabo8040058)
Supplement: Supplementary file 1 [file metabolites-08-00058-s001.pdf]

Supplementary Information

Article

Identifying Antibacterial Compounds in Black Walnuts (*Juglans nigra*) Using A Metabolomics Approach

Khanh-Van Ho [1,2], Zhentian Lei [3], Lloyd Sumner [3], Mark V. Coggeshall [4], Hsin-Yeh Hsieh [5], George C. Stewart [5], Chung-Ho Lin [1,*]

1 The Center for Agroforestry, School of Natural Resources, University of Missouri, Columbia, MO, USA
2 Department of Food Technology, Can Tho University, Can Tho, Vietnam
3 Metabolomics Center, University of Missouri, Columbia, MO, USA
4 U. S. Northern Research Station, USDA-Forest Service, West Lafayette, IN, USA
5 Department of Veterinary Pathobiology, MU Bond Life Sciences Center, Columbia, MO, USA
* Corresponding author: linchu@missouri.edu

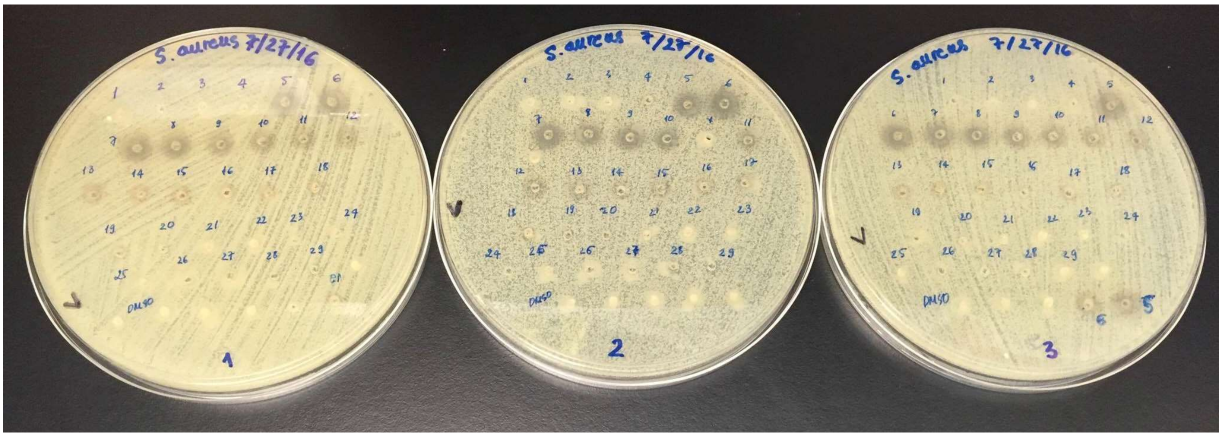

Supplementary Figure 1. Zone of inhibition of 29 Mystry fractions (out of 46) from column chromatography. 1-29: fraction 1 through fraction 29.

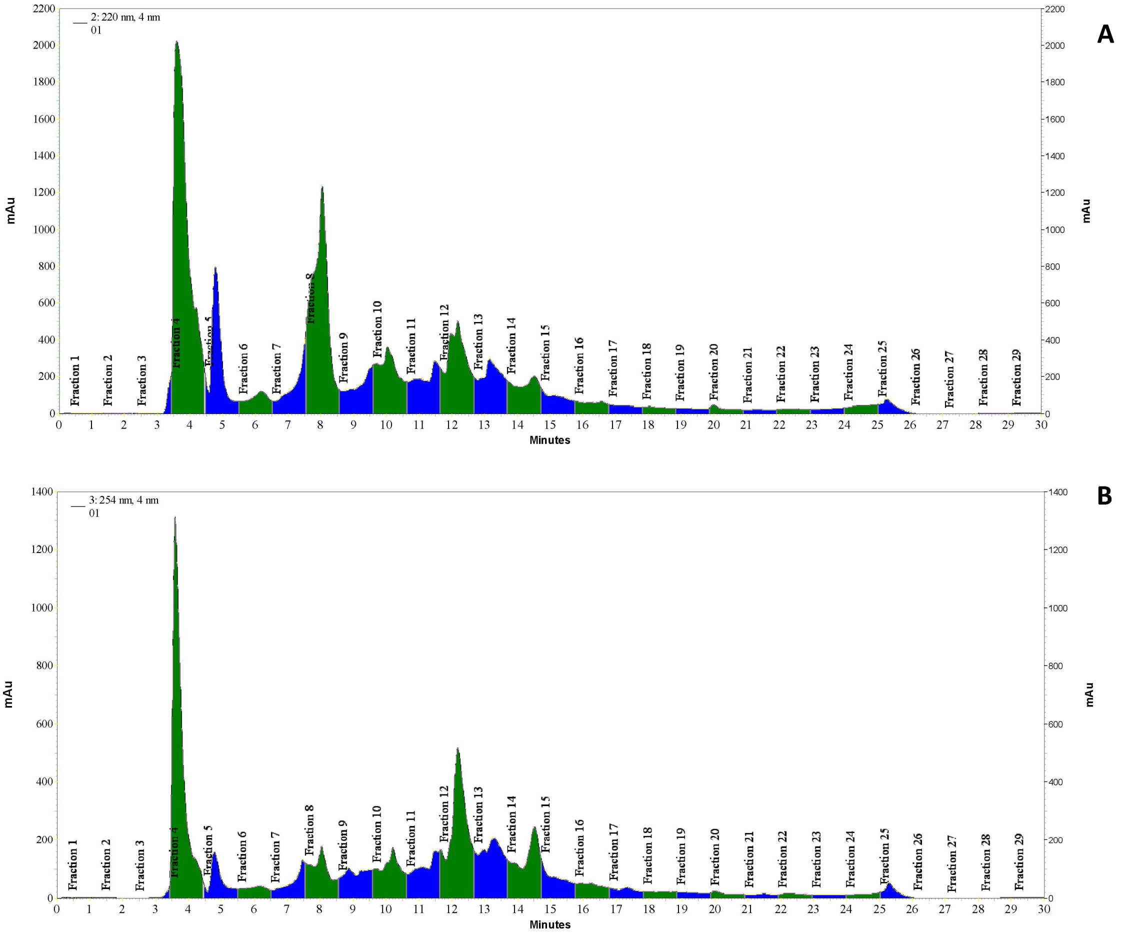

Supplementary Figure 2. Shimadzu UV Chromatogram of HPLC sub-fraction F14 at 220 nm (A) and 254 nm (B) generated from SPD-10Avp photodiode array detector.

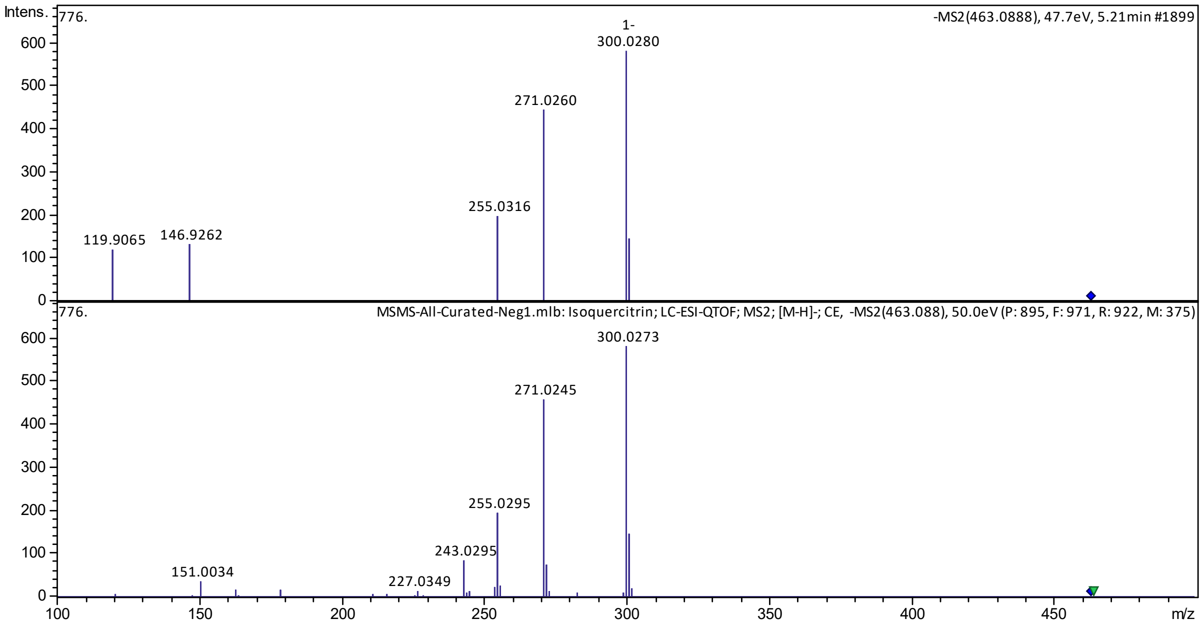
Intens.
776.
-MS2(463.0888), 47.7eV, 5.21min #1899
1-
300.0280
271.0260
255.0316
119.9065
146.9262
776.
MSMS-All-Curated-Neg1.mlb: Isoquercitrin; LC-ESI-QTOF; MS2; [M-H]-; CE, -MS2(463.088), 50.0eV (P: 895, F: 971, R: 922, M: 375)
300.0273
271.0245
255.0295
243.0295
151.0034
227.0349
0
100
200
300
400
500
600
100
150
200
250
300
350
400
450
m/z

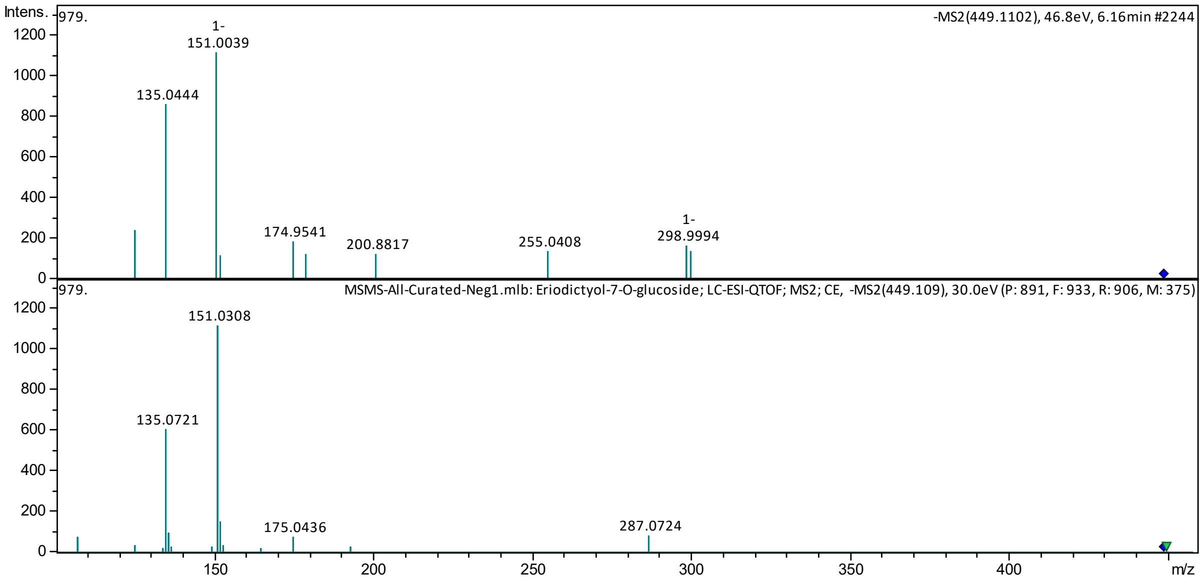
Intens.
979.
-MS2(449.1102), 46.8eV, 6.16min #2244
1-
151.0039
135.0444
174.9541
200.8817
255.0408
1-
298.9994
979.
MSMS-All-Curated-Neg1.mlb: Eriodictyol-7-O-glucoside; LC-ESI-QTOF; MS2; CE, -MS2(449.109), 30.0eV (P: 891, F: 933, R: 906, M: 375)
151.0308
135.0721
175.0436
287.0724
0
200
400
600
800
1000
1200
150
200
250
300
350
400
m/z

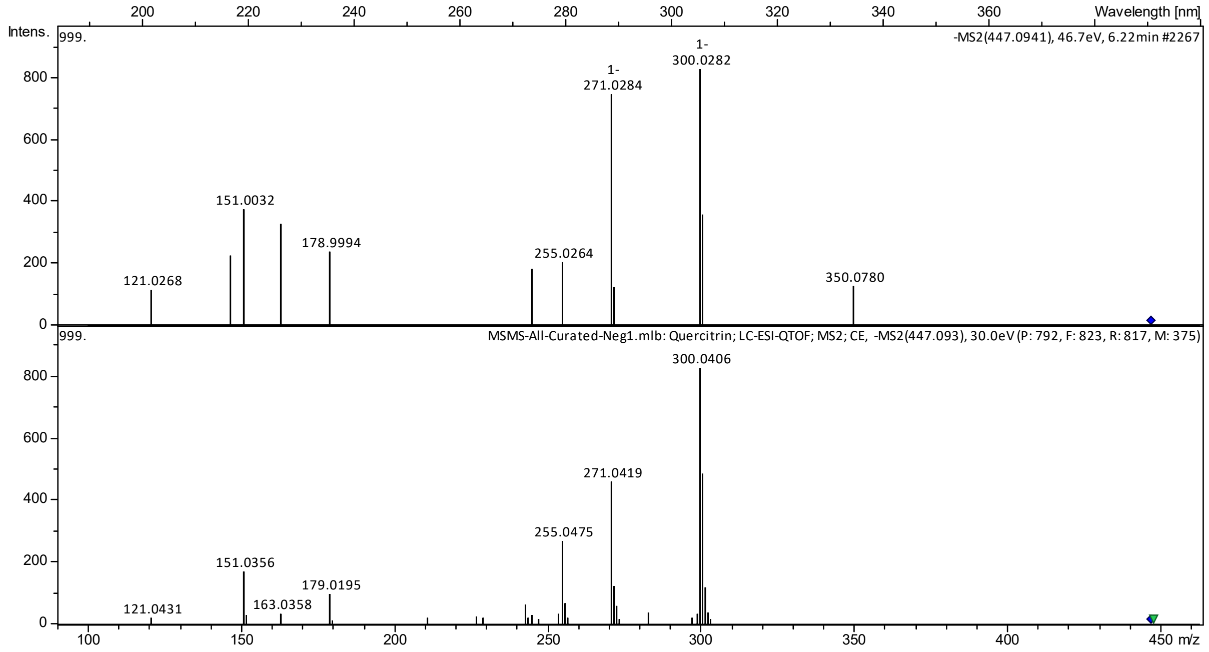

Supplementary Figure 3. Head-to-tail spectral comparisons between the experimental MS/MS spectra and the referenced library MS/MS spectra. (A) Peak 1: Isoquercitrin, m/z of 463.0888, retention time (rt) at 5.21 min; (B) Peak 2:; (C) Peak 3: Eriodictyol-7-O-glucoside, m/z of 449.1102, rt at 6.15 min; (D) Peak 4: Quercitrin, m/z of 477.0941, rt at 6.26 min; (E) Peak 5: Azelaic acid, m/z of 187.0977, rt at 6.58 min; (F) Peak 6: Glansreginin A, m/z of 592.2043, rt at 7.18 min.
